# Improved scFv Anti-LOX-1 Binding Activity by Fusion with LOX-1-Binding Peptides

**DOI:** 10.1155/2017/8946935

**Published:** 2017-09-28

**Authors:** Wei Hu, Qiuhong Xie, Hongyu Xiang

**Affiliations:** ^1^School of Life Science, Jilin University, Changchun, Jilin 130012, China; ^2^National Engineering Laboratory for AIDS Vaccine, School of Life Science, Jilin University, Changchun, Jilin 130012, China; ^3^Key Laboratory for Molecular Enzymology and Engineering, The Ministry of Education, Jilin University, Changchun, Jilin 130012, China

## Abstract

The oxidized low-density lipoprotein receptor-1 (LOX-1) targeted single-chain variable fragment (scFvs) is a promising molecule for the targeted delivery of imaging and therapeutic molecules of atherosclerotic diseases; however, its applications are limited by the inherent low antigen affinity. In this study, the three-dimensional (3D) model of the anti-LOX-1 scFv was constructed and its docking with the LOX-1 protein was developed. To improve the LOX-1-binding activity, the anti-LOX-1 scFv was designed to fuse with one of three LOX-1-binding heptapeptides, LTPATAI, FQTPPQL, and LSIPPKA, at its N-terminus and C-terminus and in the linker region, which have different LOX-1-binding interfaces with the anti-LOX-1 scFv analyzed by an array of computational approaches. These scFv/peptide fusions were constructed, successfully expressed in* Brevibacillus choshinensis* hosts, and purified by a two-step column purification process. The antigen binding activity, structural characteristics, thermal stability, and stability in serum of these fusion proteins were examined. Results showed that the scFv with N-terminal fusing peptides proteins demonstrated increased LOX-1-binding activity without decrease in stability. These findings will help increase the application efficacy of LOX-1 targeting scFv in LOX-1-based therapy.

## 1. Introduction

LOX-1, the major endothelial oxidized low-density lipoprotein (oxLDL) receptor-1 [1], is undetectable under healthy conditions but is highly expressed in atherogenic settings and atherosclerotic lesions [2]. LOX-1 has been shown to be a specific biomarker for atherosclerosis-related diseases [3], and LOX-1 targeting therapies present a reliable strategy for the management of these diseases. Indeed, LOX-1-mediated proatherogenic effects can be inhibited by anti-LOX-1 monoclonal antibodies (MAbs) [4], and LOX-1 can be utilized for plaque imaging with MAbs as well as for selective delivery of antiatherosclerotic agents [5]. However, the application of the intact MAb molecules is limited by potential side effects and high production costs [6]. Their large size also limits their ability to penetrate the diseased areas. In addition, binding of the fragment crystallizable (Fc) domain of the MAbs to Fc receptors on the cell surface limits the circulation and mobility of the whole molecules [7].

As an alternative to MAbs, antibody fragments such as single-chain antibody fragments (scFvs) are increasingly being used in diagnostic and therapeutic applications, due to their specific binding affinity to antigens, superior biodistribution, low immunogenicity, low cost, and high modifiability [8]. In our previous study, an anti-LOX-1 scFv was successfully produced in the* Brevibacillus choshinensis (B. choshinensis)* host and showed specific affinity for the human LOX-1 protein [9]. We believe that it has a great potential for immunodiagnostics, drug delivery, and immunotherapy. However, despite the great potential of scFv molecules, few antibody fragments possess ideal biophysical properties for medical applications [10] because of inherent low antigen affinity.

Peptides, an efficient small molecular vector, are promising candidates for delivery of therapeutic proteins* in vitro* and* in vivo* [11]. In previous study, LOX-1-binding peptides LTPATAI, FQTPPQL, and LSIPPKA were identified and proved to be promising candidates for the selective targeting of viral and nonviral gene transfer vectors to endothelial cells expressing the LOX-1 receptor* in vitro* and* in vivo* [12]. Advantages of peptides are that peptides (i) can bind with high affinity to protein up to 3.5 nM [13] and (ii) in general have low toxicity and low immunogenicity, (iii) more efficient tissue penetration, and generally lower production costs [14]. Peptides can also have some disadvantages, including their potentially poor pharmacokinetic parameters and moderate specificity because of the small size and antigen recognizing mode.

In this study, to improve the application efficacy, the anti-LOX-1 scFv and LOX-1-binding peptides were designed to fuse together. Before that, the molecular docking of the scFv and peptides with LOX-1 were constructed, respectively, and the binding interfaces were analyzed using an array of computational approaches, and if the peptides compete with scFv for LOX-1 binding, the fusions would inhibit but not enhance binding. Results showed that they have different LOX-1-binding sites, and there is a chance to improve the LOX-1-binding activity by connecting the two parts together. The anti-LOX-1 scFv was designed to fuse with one of three LOX-1-binding heptapeptides, LTPATAI, FQTPPQL, and LSIPPKA, at its N-terminus and C-terminus or in the linker region. These scFv/peptide fusions were constructed, expressed in the* B. choshinensis *host, and purified by two-step column chromatography. The antigen binding activity, structure characteristics, thermal stability, and stability in serum of these fusion proteins were examined to evaluate whether they are suitable for the diagnosis and treatment of atherosclerosis-related diseases.

## 2. Materials and Methods

### 2.1. Materials

*B. choshinensis* SP3 (Takara, Japan) was used for protein expression. The* B. choshinensis-E. coli* shuttle vector pNCMO2 (Takara) was used for expression in* B. choshinensis*. A site-directed mutagenesis kit was purchased from Stratagene (Shanghai, China). Recombinant human LOX-1 protein was purchased from Sino Biological Inc., China. Anti-His tag antibody and HRP-conjugated goat anti-mouse IgG were purchased from TransGen Biotech (Beijing, China). Bovine serum albumin (BSA) and neomycin were purchased from Sigma-Aldrich, UK. TM medium with modifications (3% glucose, 2.2% polypeptone, 0.8% meat beef extract, 0.2% yeast extract, 0.001% FeSO_4_·7H_2_O, 0.001% MnSO_4_·4H_2_O, and 0.0001% ZnSO_4_·7H_2_O) was used to culture the* B. choshinensis* strains.

### 2.2. Molecular Simulation Study

Phyre2 was applied to build the 3D structure of the anti-LOX-1 scFv (http://www.sbg.bio.ic.ac.uk/phyre2/html/page.cgi?id=index) [15]. For the docking study, the crystal structure of the LOX-1 protein (ID: 1YPQ) downloaded from the Protein Data Bank (PDB) was used. The structures generated were refined by the GalaxyWEB server (http://galaxy.seoklab.org/) [16]. Docking of the anti-LOX-1 scFv to the LOX-1 protein was performed using the automated initial stage docking algorithm implemented by ZDOCK (http://zdock.umassmed.edu/) [17]. Docking of the LOX-1-binding peptides to the LOX-1 protein was performed using CABS-dock (http://biocomp.chem.uw.edu.pl/CABSdock/) [18]. For all the original models, energy minimization and 30 ns molecular dynamic simulations were performed using standard GROMOS96 43a2 force field for model refinements [19]. The binding analysis of the antigen-ligand complexes was analyzed using Discovery Studio 2.5 (Accelrys Software Inc.), which was also used to display the final pictures. mCSM-AB, a web server (http://biosig.unimelb.edu.au/mcsm_ab/), was used for predicting docking complexes affinity changes upon mutation [20].

### 2.3. Construction of the scFv/Peptide Expression Plasmids

The anti-LOX-1 scFv with a C-terminal Myc peptide and a polyhistidine (6xHis) metal-binding tag was synthesized according to a previous study [9] and cloned into a pUC19 vector (pUC19-scFv). Three LOX-1-binding peptides LTPATAI, FQTPPQL, and LSIPPKA were designed to fuse at the N-terminus and C-terminus or to substitute the amino acids GGGGS in the middle of the linker region of the scFv. To construct the scFv with the peptide fusion at the linker region, site-directed mutagenesis was performed by PCR using the primer sets MLTP/FQT/LSI-F and MLTP/FQT/LSI-R. To construct the scFv with the peptide fusion at the C-terminus between 6xHis and Myc tags, site-directed mutagenesis was performed by PCR with the primer sets CLTP/FQT/LSI-F and CLTP/FQT/LSI-R. To construct the scFv with the peptide fusion at the N-terminus, we fused the peptide and a linker peptide to the N-terminus of the scFv with two overlap extension PCRs with the forward primer NLTP/FQT/LSI-F1/2 and the same reverse primer scFv-R. To generate the expression plasmids, these PCR products were digested with restriction enzymes* Sal*I and* EcoR*I and cloned into the pNCMO2 vector digested with the corresponding restriction enzymes. The primers used in the cloning are listed in Table S1 (in Supplementary Material available online at https://doi.org/10.1155/2017/8946935).

### 2.4. Expression and Purification of the scFv/Peptide Fusion Proteins

*B. choshinensis *SP3 cells harboring the expression plasmids were cultured at 30°C in TM media containing 10 mg/L neomycin (Sigma-Aldrich, China). The supernatants were collected by centrifugation at 5400 ×g for 20 min at 4°C and subsequently loaded onto a 5-mL HisTrap HP column (GE Healthcare, USA), and the column was washed with PBS buffer (2 mM KH_2_PO_4_, 18 mM Na_2_HPO_4_·12H_2_O, pH 7.4) containing 0.2 M NaCl and 25 mM imidazole. The bound proteins were eluted by gradually increasing the imidazole concentration to 0.5 M. The peak fractions of the HisTrap HP column were pooled and immediately applied to a HiLoad 26/60 Superdex 200 pg (GE Healthcare). The purified scFv was analyzed by reducing 10% SDS-PAGE and western-blotting, and the concentration was measured using the BCA protein assay kit (Pierce, USA).

### 2.5. Affinity Analyzed by Noncompetitive ELISA

Noncompetitive ELISA was used to determine the affinity of the scFv/peptide fusion proteins to human recombinant LOX-1. Briefly, twenty micrograms of recombinant human LOX-1 protein was biotinylated at a 1 : 1 ratio with EZ-Link Sulfo-NHS-Biotin (Pierce). A streptavidin coated 96-well plate was coated with the biotinylated LOX-1 protein at 4°C overnight and then incubated with the purified anti-LOX-1 scFv or scFv/peptide fusion proteins or incubated with 2% BSA set as the negative control. Subsequently, the plate was sequentially incubated with the anti-His tag antibody and HRP-conjugated goat anti-mouse IgG, followed by the addition of tetramethylbenzidine (TMB) and measurement with a microplate reader (Thermo Labsystems) at 450 nm [21].

### 2.6. Far-UV CD Spectral Analysis

Far-UV CD spectral measurement was performed as described previously [22]. The CD spectra in the range of 195–240 nm were determined on a JASCO J-715 spectropolarimeter (Jasco, Tokyo, Japan) at room temperature. Purified antibody proteins at 0.5 g/L in PBS (pH 7.4) were used for the CD spectral analysis. Four scans were accumulated at a scan rate of 10 nm/min and a response time of 4 s using the path-length of the cell (0.2 mm). For thermal stability analysis, the changes in mean residue ellipticities at 218 nm with increasing temperatures were monitored, and the transition point was estimated.

### 2.7. Intrinsic Fluorescence Spectroscopy

The intrinsic fluorescence of the antibody samples was measured with the RF-5301 fluorescence spectrophotometer (Shimadzu, Japan) using a 1.0 cm quartz cuvette. The fluorescence emission spectra of the antibody samples at a concentration of 0.1 g/L were monitored at a scanning rate of 1 nm. The emission spectra in the range of 300–400 nm were recorded using the excitation wavelength of 295 nm. The scans were done in triplicate, and the average results are presented [23].

### 2.8. Serum Stability* In Vitro*

Five micrograms of the purified antibody samples was added to 1.0 mL of 50% mouse serum and incubated at 37°C for up to 72 h. An aliquot (100 *μ*L) of the mixture was taken out at each time point (0 h, 24 h, 48 h, and 72 h) and stored at −80°C. The sample at 0 h was set as a control. After collection, the samples were thawed on ice and analyzed for LOX-1 binding activity as previously described [24].

## 3. Results and Discussion

### 3.1. Homology Modeling of the Anti-LOX-1 scFv

**Figure 1:**
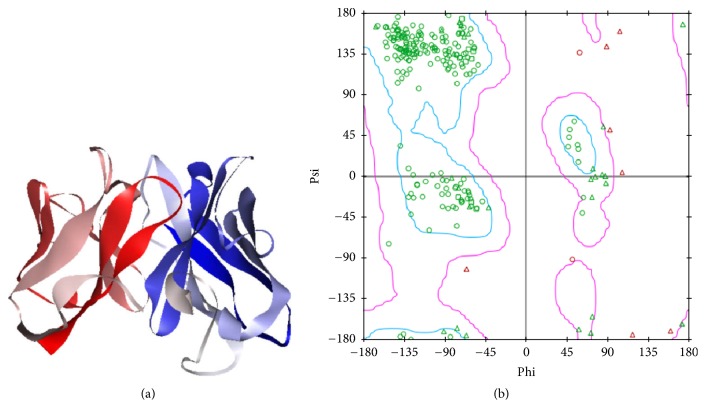
Homology modeling of the anti-LOX-1 scFv. (a) The 3D structure of the anti-LOX-1 scFv. (b) Ramachandran plot for the anti-LOX-1 scFv prepared using Discovery Studio.

Homology or comparative modeling of a protein is a method of structure prediction based on amino acid sequence similarity to closely related known structures [25]. The 3D structure of the anti-LOX-1 scFv was built by Phyre2, a suite of tools available on the web to predict and analyze protein structure, function, and mutations [15], and refined by GalaxyWEB server, which can detect unreliable regions and perform ab initio modeling to improve models [16]. Five refined models (Table S2) were constructed and the model with the best quality was selected (Figure 1(a)) and was subjected to energy minimization and 30 ns molecular dynamic simulations and checked by Procheck. The Ramachandran plot for the anti-LOX-1 scFv (Figure 1(b)) showed all residues within the core and generously allowed region. The validation of the model structure shows that the stereochemical geometry as well as the overall structural geometry of the models is good and can be further used to study interactions with ligand proteins [26].

### 3.2. The scFv and Peptides Have Different Binding Interfaces with the LOX-1 Protein

**Figure 2:**
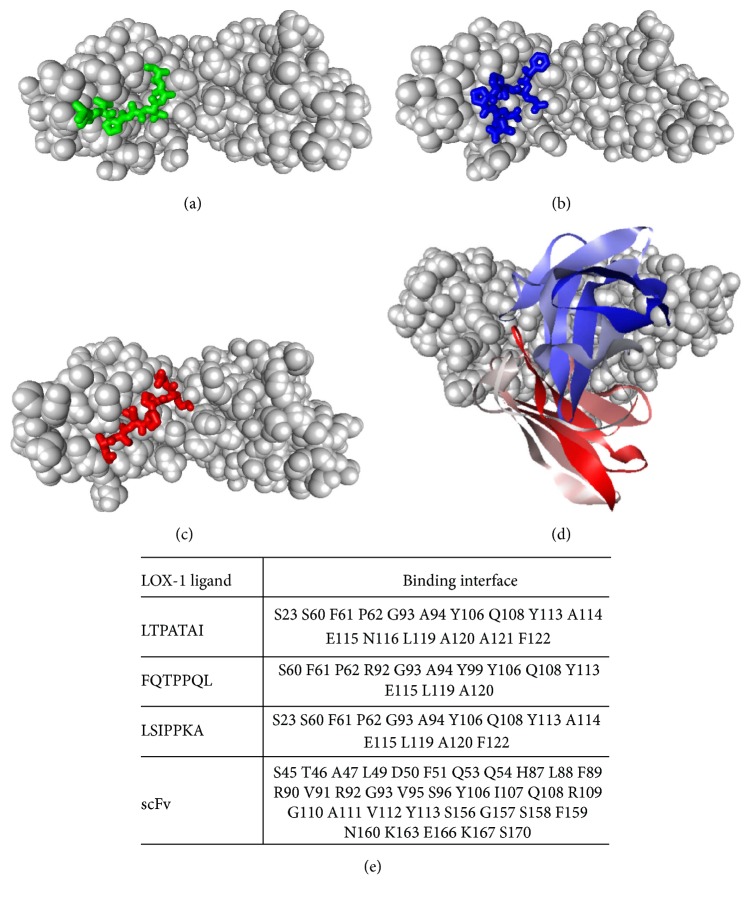
Molecular docking analysis. Docking of the LOX-1-binding peptide: (a) LTPATAI (green), (b) FQTPPQL (blue), and (c) LSIPPKA (red) with the LOX-1 protein developed by CABS-dock. (d) Docking of the anti-LOX-1 scFv (N-terminal to C-terminal was marked with gradient color blue to red) with the LOX-1 (gray) protein developed by ZDOCK. (e) Amino acids of the binding interfaces of LOX-1 with the anti-LOX-1 scFv and three LOX-1-binding peptides, respectively.

The molecular docking of the three LOX-1-binding peptides with LOX-1 was developed by CABS-dock (Figures 2(a), 2(b), and 2(c)), respectively. CABS-dock allows highly efficient modeling of full peptide flexibility and significant flexibility of a protein receptor. During CABS-dock docking, the peptide folding and binding process is explicitly simulated and no information about the peptide binding site or its structure is used [27]. The docking of anti-LOX-1 scFv with the LOX-1 protein was developed by ZDOCK (Figure 2(d)). The rigid-body protein-protein docking program ZDOCK uses the Fast Fourier Transform algorithm to enable an efficient global docking search on a 3D grid and utilizes a combination of shape complementarity, electrostatics, and statistical potential terms for scoring. ZDOCK achieves high predictive accuracy on protein-protein docking benchmarks [28].

After molecular dynamic simulations, these docking complexes were subjected to the binding analysis. To identify the key residues that drive the interaction between LOX-1 and its ligands, the binding interfaces of the LOX-1 protein interacted with the scFv or three peptides were analyzed by Discovery Studio 2.5 [29] and displayed in Figure 2(e). Results indicated that the three peptides have similar binding sites with the LOX-1 protein, which are different from that of the anti-LOX-1 scFv. To verify that the residues in the binding interfaces of the three peptides interacted with LOX-1 cannot affect the binding affinity of the scFv with the LOX-1 protein, the scFv-LOX-1 docking complexes affinity changes upon the mutation of these residues were analyzed by mCSM-AB server. Results showed that the mutation of these resides plays a minor effect on the scFv-LOX-1 binding affinity, indicating that the three peptides have a little chance to disturb the binding of the anti-LOX-1 scFv with the LOX-1 protein (Table S3).

### 3.3. Construction and Production of the Anti-LOX-1 scFv/Peptide Fusion Proteins

**Figure 3:**
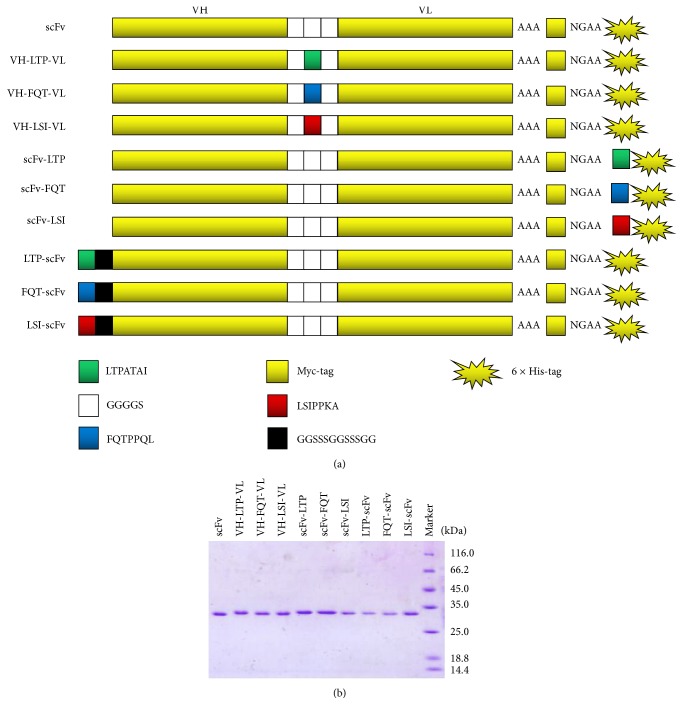
Production of the anti-LOX-1 scFv/peptide fusion proteins. (a) Schematic graphs of the fusion proteins. (b) Analysis of the purified fusion proteins by 10% SDS-PAGE.

As the anti-LOX-1 scFv and three LOX-1-binding peptides have different binding sites with the LOX-1 protein, there is a possibility of improving the LOX-1 binding activity by fusing the two parts together. As the binding region (CDR) and frame region (FR) encoded by the variable domain genes play vital roles in the activity and stability of the scFv molecule [30], to avoid disrupting these regions in the parental scFv, the three LOX-1-binding peptides were designed to fuse at the N-terminus of the starting scFv with a linker peptide (called LTP-scFv, FQT-scFv, and LSI-scFv) or at the C-terminus between Myc and 6×His tags (called scFv-LTP, scFv-FQT, and scFv-LSI) or replace the amino acids GGGGS in the middle of the linker of the starting scFv (called VH-LTP-VL, VH-FQT-VL, and VH-LSI-VL). The schematic representations of these scFv/peptide fusions are displayed in Figure 3(a).

The scFv/peptide expression plasmids were transformed into the* B. choshinensis* hosts for expression and subsequent purification with the HisTrap HP column and subsequent gel filtration. The purified scFv/peptide fusion proteins were analyzed by SDS-PAGE (Figure 3(b)). All the fusion proteins were secreted in soluble forms with high yields, indicating that these fusion proteins could be correctly folded, and the fusing peptides did not affect the expression and purification since these steps were performed in the same manner as that for the parental scFv.

### 3.4. LOX-1 Binding Activity of the scFv/Peptide Fusion Proteins

**Figure 4:**
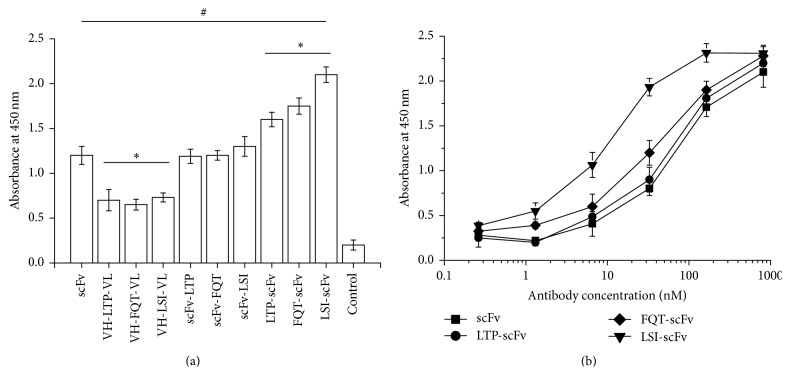
Binding activity of the anti-LOX-1 scFv/peptide fusion proteins against immobilized human LOX-1 protein. (a) LOX-1 binding ability changes of the fusion proteins analyzed by ELISA. (b) Dose-dependent responses. The values displayed are the average results of three experiments. “#” indicates *P* < 0.01 versus the control group. “*∗*” indicates *P* < 0.01 versus the scFv group.

The binding activity of the scFv/peptide fusion protein against the human LOX-1 protein was estimated by ELISA and the average results from three experiments were displayed in Figure 4. Compared with the parental scFv, the fusion proteins VH-LTP-VL, VH-FQT-VL, and VH-LSI-VL showed a reduction in LOX-1-binding activity; the fusion proteins scFv-LTP, scFv-FQT, and scFv-LSI showed no obvious changes; the fusion proteins LTP-scFv, FQT-scFv, and LSI-scFv showed obviously increased LOX-1 binding activity (Figure 4(a)). To further verify the LOX-1 binding activity of the scFv with N-terminal fusing peptides, the EC50 values were calculated (Figure 4(b)). The LOX-1 binding activity of the antibody proteins at different concentrations was tested, and the data was analyzed by the software of OriginPro 8. The EC50 was set as the antibody concentration that generated 50% maximum response at OD450. Obviously, the EC50 values of the scFv/peptide fusion proteins LTP-scFv, FQT-scFv, and LSI-scFv were decreased more than that of the parental scFv, indicating increased binding activity.

### 3.5. Structural Characterization Analysis of the scFv/Peptide Fusion Proteins

**Figure 5:**
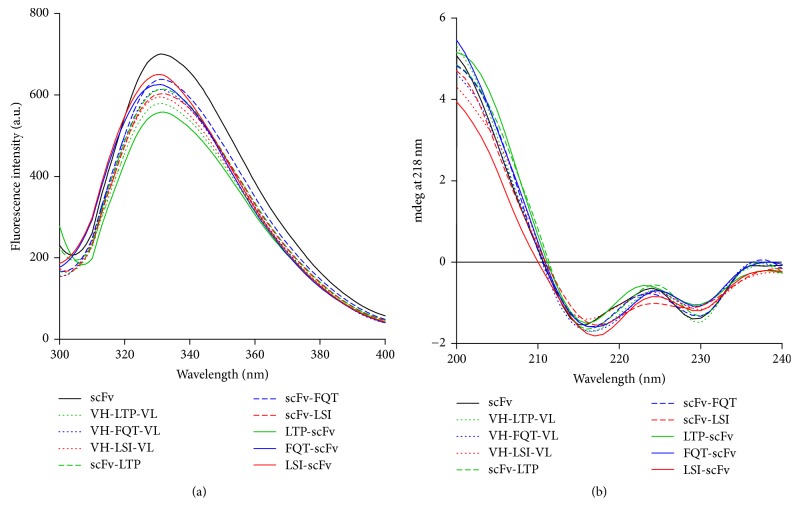
Structural analysis of the scFv/peptide fusion proteins by (a) intrinsic fluorescence spectroscopy and (b) far-UV CD spectra.

All the antibodies displayed a similar shape of the fluorescence curve with a peak between 320 and 340 nm (Figure 5(a)) and showed similar CD spectra, with a negative peak at 218 nm, consistent with the *β*-sheet found in the immunoglobulin protein folding and an obvious ellipticity minimum of the negative peak at 230 nm as described for some VL domains [31], an indication of complete folding (Figure 5(b)).

The regional secondary structure of the linker regions of the fusion proteins was predicted by GOR [32]. The predicted secondary structures of the parental scFv and fusion proteins were shown in Figure S1. When the peptides were fused at the N-terminus or C-terminus of the scFv fragment, the fusion proteins showed elongated terminus; however, the terminal fusion peptides played minor effects on the original secondary structure of the scFv fragment. The secondary structures of the fusion proteins VH-LTP-VL, VH-FQT-VL, and VH-LSI-VL changed from loop in the parental scFv to loop and rigid *β*-sheet (Table 1). It is likely that the partial rigid *β*-sheet affected the flexibility of the linker and disturbed the interactions between the VH and VL domains and thus decreased the antigen affinity of these fusion proteins. Previous studies have indicated that the flexibility of the linker plays an important role in maintaining the bioactivity of the fusion protein [33], and the linkers (GGGGS)*n* have proven to be flexible and have often been used in antibody engineering, including the construction of scFv molecules by joining VL and VH domains [34].

### 3.6. Stability Analysis of the scFv/Peptide Fusion Proteins

**Figure 6:**
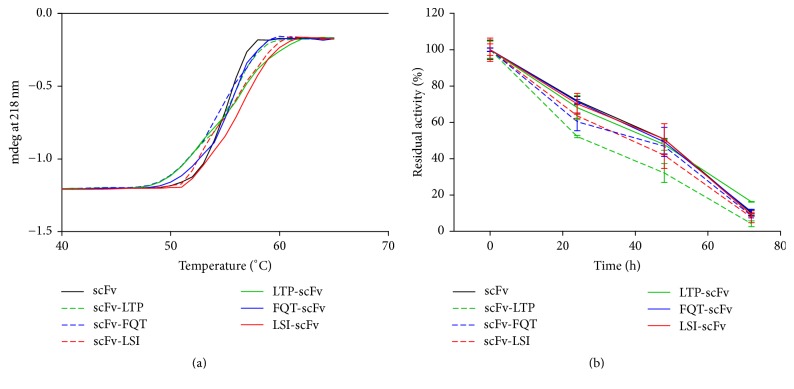
Stability of the scFv/peptide fusion proteins. (a) Changes in the ellipticity at 218 nm with the increase of temperatures. (b) Serum stability of the fusion proteins.

To investigate whether the fused peptides affected the stability of the parental scFv, we analyzed the thermal stability of these scFv/peptide fusion proteins using CD spectroscopy. Changes in the secondary structure during thermal unfolding were determined by far-UV CD spectroscopy. Since all the fusion proteins were mainly in *β*-sheet conformations with a typical 218 minimum ellipticity, the temperature-induced changes of ellipticity at 218 nm allowed us to evaluate the structural changes of the proteins with the increasing temperatures (Figure 6(a)). The changes in the CD data were best-fitted by a two-state process, and the *Tm* values of all the antibody fusion proteins were similar.

Testing the stability of the engineered antibody fragments in serum is critical in determining their potential application* in vivo*. The antibody samples were incubated in 50% mouse serum for 0 (control) to 3 days at 37°C. The binding activity of the antibody samples incubated in serum for 0 h was set as 100%, and the residual activity was calculated. Every experiment was done in triplicate, and the average results are presented (Figure 6(b)). The scFv with N-terminal fusing peptides proteins showed similar serum stability with the parental scFv, while the one with C-terminal fusing peptides proteins displayed obvious decrease in serum stability.

## 4. Conclusion

As for clinical applications, scFvs must have sufficient affinity for the target antigens; failure to meet this requirement results in insufficient enrichment of the scFv molecules at the diseased regions, thus hampering clinical applications. The aim of this study is to improve the antigen affinity of the anti-LOX-1 scFv. Antibody fragments and peptides are two widely used active targeting molecules, both of which are characterized by small molecular weight, easy production, and good modifiability. There is a possibility of constructing a fusion molecule with improved properties by connecting the two parts together. To prove this speculation, in this study, the anti-LOX-1 scFv was designed to fuse with one of three LOX-1-binding heptapeptides LTPATAI, FQTPPQL, and LSIPPKA, which have different LOX-1 binding interfaces with the anti-LOX-1 scFv analyzed by a series of computational approaches. Compared with the parental scFv, the scFv with N-terminal fusing peptides proteins were demonstrated increased LOX-1-binding activity without decrease in stability. This study not only lays a foundation for clinical application of the anti-LOX-1 scFv in LOX-1-based diagnosis and therapy but also provides valuable information for the engineering of the scFv fragments.

## Supplementary Material

Table S1. Primers used in this study.
Table S2. Five models of the anti-LOX-1 scFv refined by GalaxyWEB.
Table S3. Changes in Gibbs free energy of binding of scFv-LOX-1 docking complexes upon alanine mutation.
Fig S1. Secondary structures predicted.

## Figures and Tables

**Table 1:** Secondary structure prediction of the antibody proteins analyzed by GOR.

Antibody	Secondary structure prediction
scFv	– GGGGSGGGGSGGGGS –
	– CCCCCCCCCCCCCCC –
VH-LTP-VL	– GGGGSLTPATAIGGGGS –
	– CCCCCCEEEEEECCCCC –
VH-FQT-VL	– GGGGSFQTPPQLGGGGS –
	– CCCCCCEECCCCCCCCC –
VH-LSI-VL	– GGGGSLSIPPKAGGGGS –
	– CCCCCCEEECCCCCCCC –

E and C indicate *β*-sheet and loop, respectively.
