# Supplementary material for: Improved scFv Anti-LOX-1 Binding Activity by Fusion with LOX-1-Binding Peptides
Source: Biomed Res Int. 2017 Sep 28;2017:8946935. doi: 10.1155/2017/8946935 (PMC5637825; doi:10.1155/2017/8946935)

**Table S1.** Primers used in this study.

| Primers | sequence 5'-3' |
| --- | --- |
| MLTP-F | GGCGGCGGTGGCTCCCTGACCCCGGCTACCGCTATCGATGTGCTGATGACTC |
| MLTP-R | GAGTCATCAGCACATCGATAGCGGTAGCCGGGGTCAGGGAGCCACCGCCGCC |
| MFQT-F | GGCGGCGGTGGCTCCTTCCAGACGCCCCCACAGCTGGATGTGCTGATGACTC |
| MFQT-R | GAGTCATCAGCACATCCAGCTGTGGGGGCGTCTGGAAGGAGCCACCGCCGCC |
| MLSI-F | GGCGGCGGTGGCTCCCTGTCCATCCCCCCTAAGGCGGATGTGCTGATGACTC |
| MLSI-R | GAGTCATCAGCACATCCGCCTTAGGGGGGATGGACAGGGAGCCACCGCCGCC |
| CLTP-F | CTGAACGGTGCTGCTCTGACCCCGGCTACCGCTATCCACCATCATCACCACC |
| CLTP-R | GGTGGTGATGATGGTGGATAGCGGTAGCCGGGGTCAGAGCAGCACCGTTCAG |
| CFQT-F | CTGAACGGTGCTGCTTTCCAGACGCCCCCACAGCTGCACCATCATCACCACC |
| CFQT-R | GGTGGTGATGATGGTGCAGCTGTGGGGGCGTCTGGAAAGCAGCACCGTTCAG |
| CLSI-F | CTGAACGGTGCTGCTCTGTCCATCCCCCCTAAGGCGCACCATCATCACCACC |
| CLSI-R | GGTGGTGATGATGGTGCGCCTTAGGGGGGATGGACAGAGCAGCACCGTTCAG |
| NLTP-F1 | GGGTCGACCTGACCCCGGCTACCGCTATCGGCGGCTCCAGCTCCGGCGGTTCC |
| NLTP-F2 | GGGTCGACTCCAGCTCCGGCGGTTCCTCCAGCGGTGGCGAAGTCAAACTGCTGGAATC |
| NFQT-F1 | GGGTCGACTTCCAGACGCCCCCACAGCTGGGCGGCTCCAGCTCCGGCGGTTCC |
| NFQT-F2 | GGGTCGACTCCAGCTCCGGCGGTTCCTCCAGCGGTGGCGAAGTCAAACTGCTGGAATC |
| NLSI-F1 | GGGTCGACCTGTCCATCCCCCCTAAGGCGGGCGGCTCCAGCTCCGGCGGTTCC |
| NLSI-F2 | GGGTCGACTCCAGCTCCGGCGGTTCCTCCAGCGGTGGCGAAGTCAAACTGCTG  GAATC |
| scFv-R | GAATTCTCATTAGTGGTGGTGATGATGGTG |

**Table S2.** Five models refined by GalaxyWEB.

| Model | GDT-HA | RMSD | MolProbity | Clash score | Poor rotamers | Rama favored |
| --- | --- | --- | --- | --- | --- | --- |
| MODEL 1 | 0.9604 | 0.399 | 2.263 | 18.8 | 1 | 91.8 |
| MODEL 2 | 0.94 | 0.429 | 2.228 | 19.3 | 0 | 93 |
| MODEL 3 | 0.9665 | 0.369 | 2.13 | 15.8 | 0 | 93.4 |
| MODEL 4 | 0.9573 | 0.411 | 2.235 | 16.4 | 0.5 | 91 |
| MODEL 5 | 0.9705 | 0.382 | 2.21 | 19.3 | 0.5 | 93.4 |

**Table S3.** Changes in Gibbs free energy of binding (ΔΔG) of scFv-LOX-1 docking complexes upon alanine mutation analyzed by mCSM-AB server.

| Residues | ΔΔG |
| --- | --- |
| S23A | -0.102 |
| S60A | -0.415 |
| F61A | -0.99 |
| P62A | -0.874 |
| G93A | -0.104 |
| A94 | － |
| Y99A | -0.898 |
| Y106A | -0.442 |
| Q108A | -0.8 |
| Y113A | -0.987 |
| A114 | － |
| E115A | -0.681 |
| N116A | -0.426 |
| L119A | -0.729 |
| A120 | － |
| A121 | － |
| F122A | -0.663 |

‘－’indicates not tested.

**Fig S1.** Secondary structures of the parental scFv and scFv/peptide fusion proteins predicted by GOR server. Red, purple and blue indicates a-helix, loop and β-sheet, respectively.


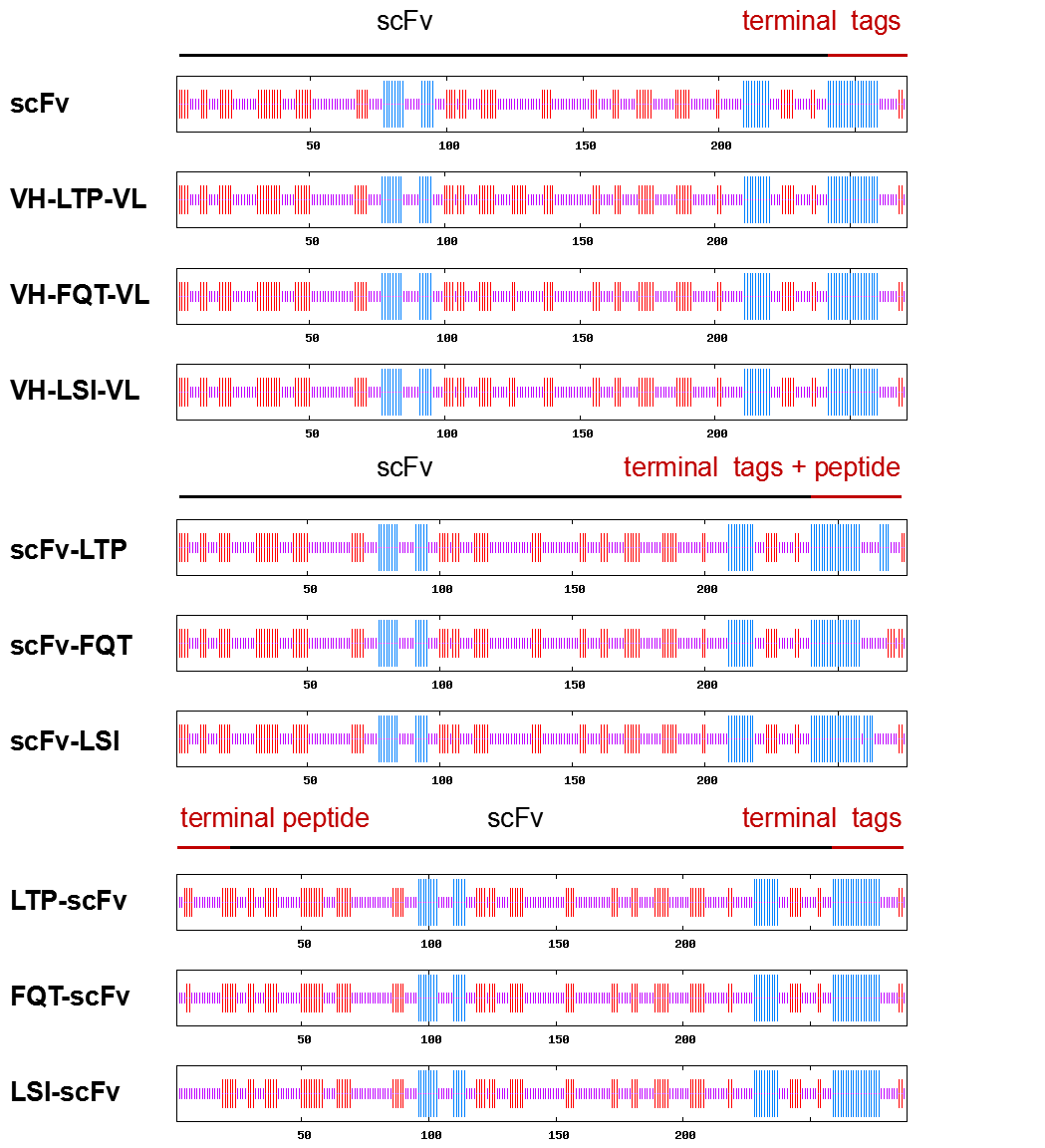

Supplement: Supplementary file 1 — Table S1. Primers used in this study. Table S2. Five models of the anti-LOX-1 scFv refined by GalaxyWEB. Table S3. Changes in Gibbs free energy of binding of scFv-LOX-1 docking complexes upon alanine mutation. Fig S1. Secondary structures predicted. [file 8946935.f1.doc]
